# Multifaceted role of ferroptosis in cardiovascular disease: Ferroptosis in cardiovascular disease

**DOI:** 10.3724/abbs.2023019

**Published:** 2023-02-23

**Authors:** Chengyi Li, Xiusong Zhu, Juxian Chen, Xiaoxi Xie, Sijia Liang, Xiu Liu, Quan Gong, Jiawei Guo

**Affiliations:** 1 Department of Immunology School of Medicine Yangtze University Jingzhou 434023 China; 2 School of Medicine Yangtze University Jingzhou 434023 China; 3 Department of Pharmacology Zhongshan School of Medicine Sun Yat-sen University Guangzhou 510080 China; 4 Department of Cardiovascular Surgery Nanfang Hospital Southern Medical University Guangzhou 510515 China; 5 Department of Pharmacology School of Medicine Yangtze University Jingzhou 434023 China

**Keywords:** cardiovascular disease, ferroptosis, glutathione peroxidase 4

## Abstract

Ferroptosis is a newly identified form of non-apoptotic cell death characterised primarily by iron-dependent lipid peroxidation. It differs morphologically, biochemically, and genetically from other forms of cell death, such as apoptosis, autophagy, and necrosis. Although the molecular mechanism underlying ferroptosis remains unclear, multiple biological processes, such as iron metabolism, lipid peroxides, and systems, such as the glutathione system and the tetrahydrobiopterin/coenzyme Q10 system, appear to be involved. While the contribution of ferroptotic mechanisms to human diseases is not clear, recent studies have identified a number of ferroptosis-related genes. Cardiovascular diseases are the main cause of death globally. In this review, we outline the progress regarding the emerging role of ferroptosis in the pathogenesis of cardiac pathophysiological conditions and the association of ferroptosis with cardiomyopathy, myocardial ischemia-reperfusion injury, heart failure, and atherosclerosis. We further summarise newly discovered ferroptotic targets for the development of therapies for cardiovascular diseases. Finally, we discuss the current challenges and future research directions in cardiovascular disease treatments.

## Introduction

Miscellaneous subroutines contribute to cell death. In 2012, a new mode of non-apoptotic cell death, referred to as ferroptosis and characterised by excessive iron-dependent lipid peroxidation, was discovered [1]. Further in-depth research has identified a variety of metabolic pathways associated with ferroptosis, such as iron metabolism, lipid peroxidation, the glutathione (GSH) system, and the tetrahydrobiopterin/coenzyme Q10 (BH4/CoQ10) system [2]. Ferroptotic cell death is the leading cause of various diseases.

In recent years, increasing evidence has indicated that ferroptosis driven by iron-dependent lipid peroxidation plays a central role in cardiovascular diseases (CVDs), such as cardiomyopathy, myocardial ischemia/reperfusion injury (MI/RI), heart failure, and atherosclerosis. Inhibition of ferroptosis in cardiomyocytes has been implicated in alleviation of myocardial damage caused at least in part by ferroptosis [ 3– 5] .

CVDs remain the leading cause of death worldwide [6], and ferroptosis provides a new perspective on CVD progression. Here, we systematically review the genes and pathways associated with ferroptosis and the role of ferroptosis in various forms of CVDs. We also discuss the potential of ferroptosis as a new target for the prevention and treatment of CVD.

## Genes and Pathways Related to Ferroptosis

In this section, we discuss the genes and pathways that regulate ferroptosis, including iron metabolism, lipid peroxidation, and the GSH and BH4/CoQ10 systems.

### Iron metabolism and ferroptosis

**Figure 1:**
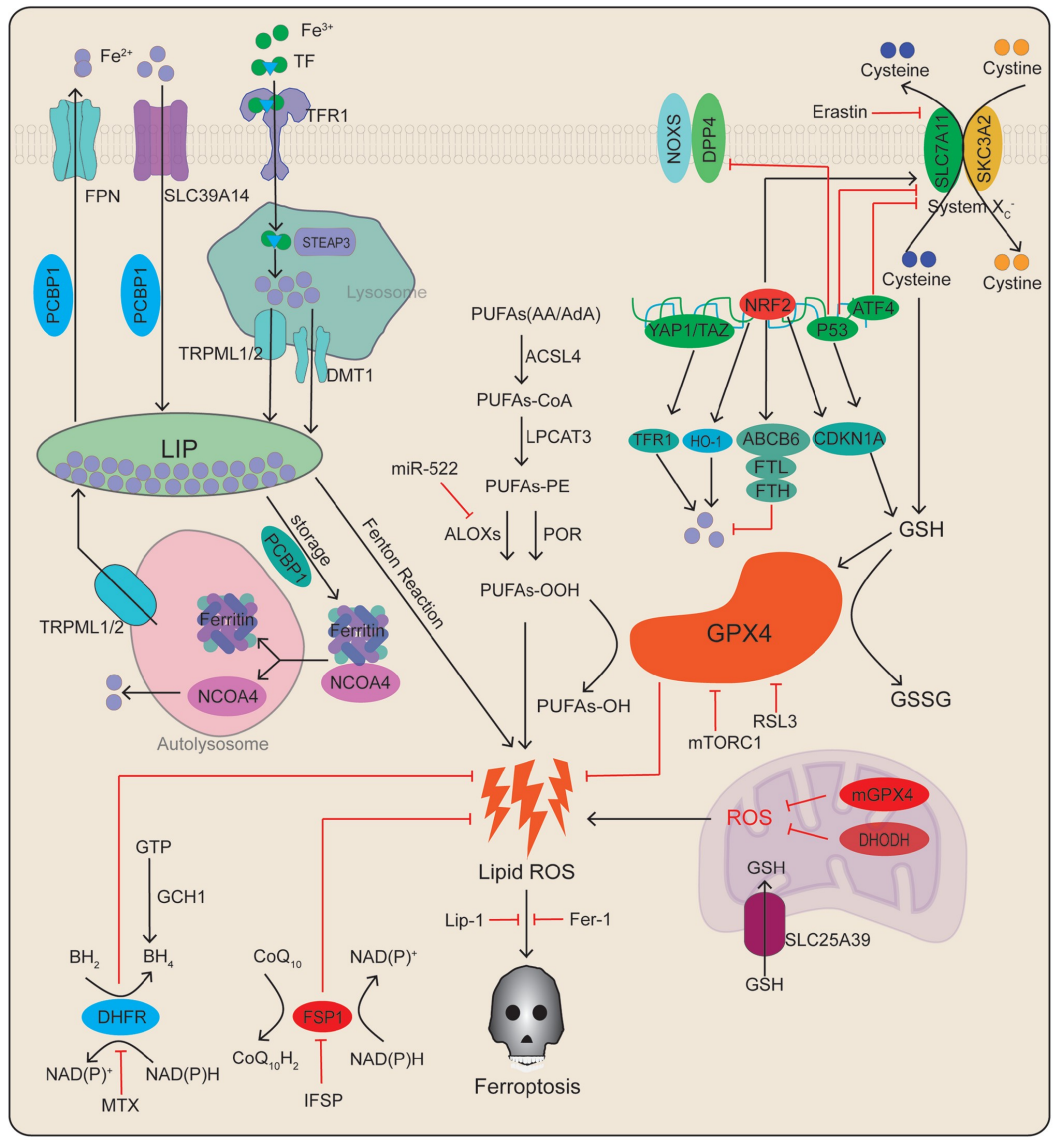
Figure 1 Basic mechanisms and regulatory pathways of ferroptosis Ferroptosis is associated with lipid peroxidation of polyunsaturated fatty acids resulted from intracellular metabolic disturbances of free Fe 2+ or glutathione peroxidation dysfunction. This diagram shows the inducers and inhibitors of ferroptosis. The arrows and blunt lines indicate promotion and inhibition, respectively, of ferroptosis. FPN, ferroportin; SLC39A14, metal transporter proteins; PCBP, poly (RC)-binding protein; LIP, labile iron pool; NCOA4, nuclear receptor coactivator 4; TF, transferrin; TFR1, transferrin receptor 1; STEAP3, six-transmembrane epithelial antigen of prostate 3; TRPML1/2, mucolipin TRP channel 1/2; DMT1, divalent metal-ion transporter 1; PUFA, polyunsaturated fatty acids; LPCAT3, lysophosphatidylcholine acyltransferase 3; ACSL4, acyl-CoA synthetase4; POR, cytochrome P450 oxidoreductase; ALOXs, lipoxygenase; GPX4, glutathione peroxidase 4; ROS, reactive oxygen species; DPP4, dipeptidyl peptidase-4; NRF2, nuclear factor erythroid 2-related factor 2; ABCB6, ATP-binding cassette subfamily B member6; ATF4, activating transcription factor 4; YAP, Yes-associated protein; FTH, ferritin heavy chain; FTL, ferritin light chain; CDKN1A, cyclin-dependent kinase inhibitor p21; TfR1, transferrin receptor1; HO-1, heme oxygenase 1; mTORC1, mechanistic target of rapamycin complex 1; GSH, glutathione; GSSG, glutathione disulfide; BH2, 7,8-dihydrobiopterin; BH4, tetrahydrobiopterin; MTX, methotrexate; NADPH, nicotinamide adenine dinucleotide phosphate; CoQ10H2, ubiquinol; FSP1, ferroptosis suppressor protein 1; DHODH, dihydroorotate dehydrogenase; mGPX4, mitochondria glutathione peroxidase 4.

Iron homeostasis triggered by ferroptosis is involved in the pathogenesis of several diseases [7]. Transferrin and transferrin receptor 1 (TFR1) play significant roles in ferroptosis progression [8]. Iron is stored intracellularly in a ferritin-bound form, but it can also be imported into cells by metal transporters (such as SLC39A14) when the iron concentration becomes excessive. Excess iron activates the Fenton reaction, which generates reactive oxygen species (ROS), leading to ferroptosis ( Figure 1). Iron is stored in an unstable iron pool in the cytoplasm by the ferritin iron storage protein complex, which comprises a ferritin light chain (FTL) and ferritin heavy chain 1 (FTH1). The iron content of cells has been shown to be significantly increased by upregulation of transferrin receptor 1, which increases iron intake, and by downregulation of FTH1 and FTL, a phenomenon that reduces iron storage [9]. These findings indicate that ferroptosis can be resulted from both increased iron intake and decreased iron stores. Furthermore, there is mounting evidence in support of the functional roles of iron and iron metabolism in the pathogenesis of ferroptosis. Erastin-induced cell death can be enhanced by exogenous iron, but it is suppressed by iron chelators, which prevent iron overload [1].

Ferritin binds to nuclear receptor coactivator 4 (NCOA4) in autophagosomes, resulting in its lysosomal degradation and iron release. Free iron is involved in the generation of ROS through the Fenton reaction. NCOA4 overexpression increases ferritin degradation and promotes erastin-induced ferroptotic death. In contrast, *NCOA4* knockout dramatically limits erastin-induced ferroptosis and lipid peroxidation by reducing ferritin degradation [10] ( Figure 1). These results suggest that ferroptosis can be induced in response to iron overload and ferritin degradation. In summary, iron metabolism is an essential factor in ferroptosis.

### Lipid peroxides and ferroptosis

Dysregulation of lipid peroxidation is one of the principal causes of ferroptosis [11]. Polyunsaturated fatty acids (PUFAs) are the main substrates of lipid peroxidation. PUFAs are positively regulated by enzymes that include acyl-CoA synthetase long chain family member 4 (ACSL4), lysophosphatidylcholine acyltransferase 3 (LPCAT3), arachidonate lipoxygenase (ALOX) isoforms, and cytochrome P450 reductase (POR) [ 12– 15] . ACSL4 catalyses the esterification between PUFAs and coenzyme A to generate PUFA-CoA. Subsequently, LPCAT3 promotes the synthesis of phospholipids, which are oxidised by ALOXs or POR [glutathione peroxidase 4 (GPX4) can inhibit this process], ultimately leading to ROS accumulation and ferroptosis [16] ( Figure 1). Studies in humans have shown that ACSL4 and LPCAT3 are crucial factors in sensitising cells to ferroptosis ( Figure 1), suggesting that lipid peroxidation triggers ferroptosis [13]. Further experiment has demonstrated that *Gpx4*/ *Acsl4* double-knockout cells are resistant to ferroptosis [12], thus providing additional evidence that ACSL4 functions as a critical determinant of ferroptosis sensitivity by acting as a lipid metabolism regulator. Phospholipids are oxidised by POR or ALOXs, and lipid peroxidation and ferroptosis are inhibited in the livers of *POR*- and *CYB5R1*-knockout mice [15]. These findings implicate POR as a potential druggable target for the development of anti-ferroptosis therapeutics. In addition, microRNA (miR)-522 in exosomes isolated by ultracentrifugation inhibits ferroptosis by targeting ALOX15 and blocking lipid ROS accumulation [14]. These findings demonstrate that ALOX is essential in regulating lipid peroxidation, a critical driver of ferroptosis ( Figure 1). Collective evidence shows that certain enzymes (such as ACSL4, LPCAT3, ALOXs, and POR) can induce ferroptosis by catalysing lipid peroxidation synthesis.

### The GSH system and ferroptosis

#### Induction of ferroptosis by suppression of system xc-

System xc- is a membrane amino acid transporter. The heterodimer is composed of a system xc-specific light chain subunit membrane transporter (SLC7A11) and a heavy chain subunit (SLC3A2) linked by disulfide bonds. System xc- regulates the exchange of extracellular cystine and intracellular glutamate. Intracellular cystine is reduced to cysteine for the synthesis of GSH and scavenging of ROS and reactive nitrogen species ( Figure 1) [17]. Inhibiting the activity of system xc- induces depletion of intracellular cysteine and GSH, leading to iron-dependent lipid peroxidation, thus resulting in cellular damage and ferroptosis.

Additionally, several transcription factors, including p53, Yes-associated protein 1 (YAP), transcriptional coactivator with PDZ-binding motif (TAZ), nuclear factor erythroid 2-related factor 2 (NRF2), and activating transcription factor 4 (ATF4), regulate SLC7A11 expression to further regulate ferroptosis ( Figure 1). Interestingly, p53 mediates the ferroptotic process via two distinct pathways. In one pathway, activation of the tumour suppressor p53 reduces cystine uptake, which in turn limits cellular antioxidant capacity by repressing SLC7A11 transcription, thereby leading to elevated lipid peroxidation and ferroptosis [ 18, 19] . In the other pathway, p53 acts as a positive regulator of ferroptosis by inhibiting dipeptidyl peptidase-4 (DPP4) [20]. YAP and TAZ regulate ferroptosis via two pathways. YAP/TAZ controls the protein stability, nuclear localisation, and transcriptional activity of ATF4, resulting in the expression of SLC7A11, thus promoting SLC7A11 transcription and preventing lipid peroxidation and ferroptosis [21]. Alternatively, they can promote ferroptosis by upregulating several ferroptosis regulators including ACSL4 and transferrin receptor (TFRC) [22]. NRF2 restrains ferroptosis by activating downstream elements, including SLC7A11, and by promoting GSH production in response to oxidative stress [23]. Activation of the p62-Keap1-NRF2 pathway reportedly prevents NRF2 degradation, and the accumulation of NRF2 has been reported to protect against ferroptosis in hepatocellular carcinoma cells ( Figure 1) [24]. These findings suggest that NRF2 plays an important role in ferroptosis. Furthermore, while overexpression of ATF4 is sufficient to inhibit ferroptosis in hepatocellular carcinoma cells, ATF4 downregulation activates ferroptosis [ 25, 26] ( Figure 1). Thus, the aforementioned transcription factors may be involved in different pathways that mediate ferroptosis.

#### Induction of ferroptosis by suppression of GPX4

GPX is a member of an evolutionarily conserved protein family that requires GSH as a cofactor to produce glutathione disulfide (GSSG) as a by-product. GPX4 is an isoform of GPX, which is a key regulator of ferroptosis. Inhibition of GPX4 can trigger ferroptosis, leading to the accumulation of lipid peroxides and, ultimately, cell death [27] ( Figure 1). RAS-selective lethal 3 (RSL3) is a ferroptosis inducer that directly targets GPX4, resulting in ROS accumulation and subsequent ferroptosis [28].

#### Mitochondrial GPX4 and dihydroorotate dehydrogenase (DHODH) restrain ferroptosis

Mitochondria are crucial organelles that function as factories for energy generation in the form of ATP via the tricarboxylic acid cycle. They are also an important source of ROS, which play an important role in maintaining cell viability. In addition, PUFAs in the lipid bilayer are more susceptible to ROS attack, leading to lipid peroxidation, which is prone to trigger ferroptosis [29]. Studies have found that the presence of two defense enzymes in the mitochondria (DHODH and mitochondrial GPX4) can counteract the damage caused by lipid peroxidation ( Figure 1). These two enzymes alleviate lipid peroxidation and ferroptosis in the mitochondria. Thus, mitochondrial GPX4 and DHODH are vital targets for regulating lipid peroxidation in mitochondria [30].

### The BH4/CoQ10 system and ferroptosis

#### Suppression of ferroptosis by the NAD(P)H/FSP1/CoQ10 system

NADPH, which is primarily produced by the pentose phosphate pathway, is a powerful reducing agent. Moreover, ferroptosis suppressor protein 1 (FSP1) uses NADPH to increase the amount of ubiquinol, the reduced form of CoQ10, which traps radicals that cause lipid peroxidation, thereby preventing oxidative lipid damage and ferroptosis [ 27, 31] . These findings suggest that the NAD(P)H/FSP1/CoQ10 system is related to metabolic processes associated with cellular lipid peroxidation reactions [27].

#### Suppression of ferroptosis by the GTP cyclohydrolase-1 (GCH1)/BH4/DHFR system

GCH1 and its metabolic derivative tetrahydrobiopterin/dihydrobiopterin (BH4/BH2) have been shown to inhibit lipid peroxidation via genome-wide activation screening. Importantly, GCH1 is a rate-limiting enzyme in biosynthesis [32]. GCH1 in cells prefabricates CoQ10 by synthesising BH4/BH2 and inhibits lipid peroxidation and ferroptosis ( Figure 1). Coenzyme reduction by panthenol inhibits lipid peroxidation and ferroptosis [27]. Recent studies have shown that GPX4 and GSH are not affected by GCH1 overexpression, suggesting that GCH1 prevents ferroptosis by inhibiting lipid peroxidation by BH4/BH2, thus acting in parallel with the canonical glutathione-based GPX4 pathway [32].

## Ferroptosis in CVDs

This section details the genes and pathways associated with ferroptosis in CVDs and potential targets for the treatment of CVDs.

### Ferroptosis and cardiomyopathy

Cardiomyopathy is a heterogeneous myocardial disease. The etiology of primary cardiomyopathy remains unclear and is mainly associated with allergies (e.g., sulfonamides), metabolic diseases (e.g., diabetes), and infections (e.g., sepsis) [ 33– 36] . Recent research has shown that some of the processes that lead to cardiomyopathy by various factors are closely related to ferroptosis. Notably, myocardial damage can be mitigated by ferroptosis inhibitors, iron-chelating agents, and small antioxidant molecules [ 37, 38] .

#### Ferroptosis and doxorubicin-induced cardiomyopathy

**Figure 2:**
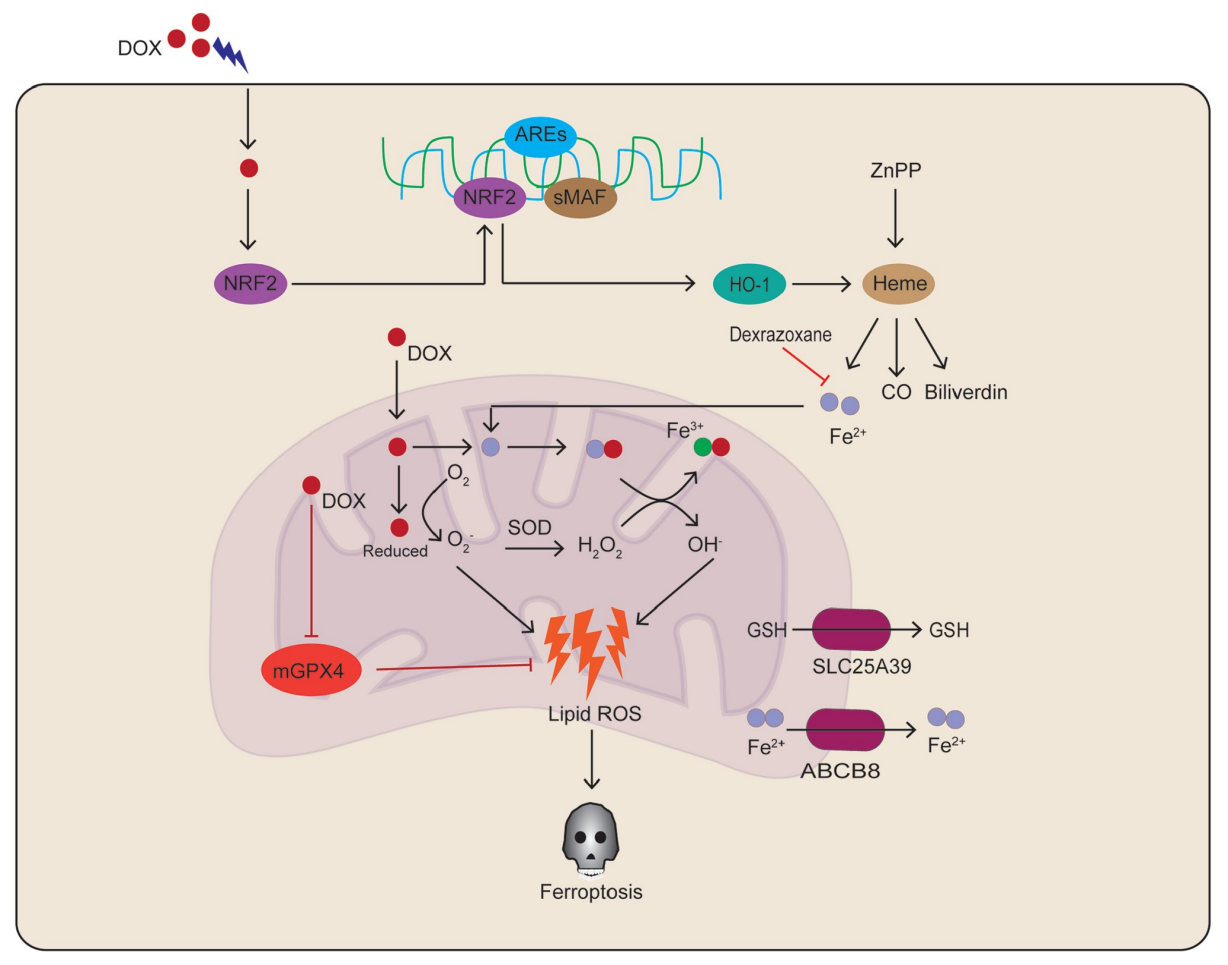
Figure 2 Doxorubicin-induced ferroptosis ultimately leads to cardiomyopathy Doxorubicin directly inhibits mitochondrial GPX4 to enhance lipid peroxidation and promote heme degradation, thereby increasing Fe 2+ levels and resulting in iron overload. mGPX4, mitochondrial glutathione peroxidase 4; SLC25A39, solute carrier family 25 member 39; ABCB8, ATP-binding cassette subfamily B member 8; DOX, doxorubicin; HO-1, heme oxygenase 1; SOD, superoxide dismutase; ZnPP, zinc protoporphyrin.

Doxorubicin (DOX) is a widely used anti-cancer chemotherapy drug. However, its application is limited because of its cardiotoxic side effects, and DOX has long been recognised as a cardiovascular risk factor [ 33, 34] . The typical side effects of DOX include dose-dependent cardiotoxicity, which causes dilated cardiomyopathy and heart failure [33] ( Figure 2). Cardiomyocytes are more vulnerable to be attacked by the cardiotoxic effects of DOX owing to iron overload via ferroptosis [ 33, 34] .

Mechanistically, the increased nuclear accumulation of NRF2 by DOX promotes the expression of HO-1, which is involved in the degradation of protoheme and release of free iron in the heart [37] ( Figure 2).

In addition, DOX has been reported to chelate Fe ^2+^ to form DOX-Fe ^2+^ complexes with the generation of OH ^–^ via the Fenton reaction [39], ultimately resulting in lipid peroxidation and cardiac damage [ 33, 40] ( Figure 2). ABC protein B8 (ABCB8) exerts its anti-ferroptotic function in mitochondria via the export of Fe ^2+^ from the mitochondria. Strikingly, overexpression of ABCB8 reverses DOX-mediated mitochondrial iron overload and cellular ROS to ameliorate cardiac injury [41] ( Figure 2). These findings suggest that DOX-induced cardiomyopathy can be prevented by lowering mitochondrial iron levels. Dexrazoxane is effective in preventing DOX-induced cardiomyopathy via the hydrolysis of free iron into active metal-chelating metabolites [37]. Ferrostatin-1 (Fer-1) significantly alleviates the accumulation of iron and the damage of disseminated intravascular coagulation *in vivo*, suggesting that Fer-1 can also prevent cardiomyocyte injury via the ferroptosis pathway [38]. Dexrazoxane and Fer-1 have the ability to attenuate acute and chronic ischemia/reperfusion injury (I/R)-induced cardiac failure [42]. This suggests that dexrazoxane and Fer-1 could be used as potential therapeutics to reduce DOX-induced cardiac injury in mice and hopefully in humans.

#### Ferroptosis and diabetic cardiomyopathy (DCM)

Diabetes mellitus is a complex metabolic disease characterised primarily by hyperglycaemia. DCM is a significant contributor to morbidity and mortality among individuals with diabetes [35]. DCM is characterised by cardiac hypertrophy and fibrosis. The pathogenesis of DCM is closely associated with mechanisms of ferroptosis regulation. Nuclear factor erythroid 2-related factor 2 (NRF2) is a key regulator of the antioxidant response. NRF2 activators inhibit ferroptosis via the AMP-activated protein kinase (AMPK)/nuclear factor erythroid 2-related factor 2 (NRF2) pathway [5]. Thus, NRF2 may provide new insights into therapeutic targets for DCM.

#### Ferroptosis and septic cardiomyopathy

Sepsis is caused by an overwhelming inflammatory response to pathogenic microorganisms. Importantly, sepsis cardiomyopathy is a common complication in patients with sepsis, and it remains one of the leading causes of death from sepsis. Sepsis cardiomyopathy is mainly characterised by the death of terminally differentiated cardiomyocytes [ 36, 43] . However, an effective treatment for septic cardiomyopathy is unavailable in current clinical practice, and further research is needed. Prostaglandin-endoperoxide synthase 2 (PTGS2, also known as cyclooxygenase-2) is a marker of ferroptosis. PTGS2 expression has been shown to be increased in patients with septic cardiomyopathy [ 36, 43] . These studies also demonstrated that lipopolysaccharide (LPS) or stimulators of interferon genes are involved in the pathological process of septic cardiomyopathy via activation of apoptosis, autophagy, pyroptosis, or necroptosis. Furthermore, the mitochondrial morphological changes induced by LPS are consistent with changes that occur in ferroptotic cells [44]. Notably, a recent study found that NCOA4 expression is enhanced in mice stimulated by LPS and that NCOA4 interacts with ferritin to promote its degradation by autophagy [45]. The release of a large amount of free iron from ferritin can activate SFXN1 located in the mitochondrial membrane. SFXN1 transports free iron from the cytoplasm to mitochondria, thereby contributing to mitochondrial iron overload and excess ROS accumulation, ultimately leading to lipid peroxidation and ferroptosis [45]. These findings in mouse models suggest that heart tissue is more sensitive to ferroptosis during septic cardiomyopathy. Therefore, targeted inhibition of ferroptosis in cardiomyocytes holds great promise as a viable treatment option for septic cardiomyopathy.

### Ferroptosis and myocardial ischemia/reperfusion injury (MI/RI)

Acute myocardial infarction is defined as myocardial necrosis caused by coronary artery ischemia and hypoxia. This type of infarction leads to MI/RI, which promotes lipid peroxidation and ferroptosis [46]. Here, we describe the relationship between MI/RI and the mechanisms involved in ferroptosis.

A recent study reported the contribution of the ferroptosis inducer erastin to ferroptosis by inducing endoplasmic reticulum stress (ERS) in rats with MI/RI. Inhibition of ERS alleviated ferroptosis as well as myocardial cell damage and MI/RI [47]. These findings suggest that ferroptosis is associated with ERS in MI/RI rat models. Cardiomyocyte injury was shown to be associated with ferroptosis and elevated levels of ERS in the MI/RI diabetic rat model by ligation of the left anterior descending coronary artery. The symptoms were prevented by further administration of Fer-1 [42]. Thus, ferroptosis in cardiomyocytes can be induced by activation of ERS during myocardial infarction, which in turn can be aggravated by ferroptosis, thus creating a vicious cycle. ERS-induced ferroptosis plays an important role in MI/RI, while inhibition of ferroptosis can protect against injury caused by MI/RI. Therefore, targeted inhibition of ferroptosis has become a strategy to improve therapeutic outcomes in patients with MI/RI.

Scavenging ROS in cardiomyocytes may attenuate the occurrence of ferroptosis [48]. In addition, the ferroptosis inhibitor lipase-1 (Lip-1), which inhibits ferroptosis by increasing GPX4 levels and decreasing ROS levels, may ameliorate ischaemia‒reperfusion-induced injury [49]. In an animal model, ROS levels were significantly increased in cardiomyocytes following MI/RI, accompanied by higher levels of p53 but lower levels of sirtuin-1 (SIRT-1) and SLC7A11. Conversely, overexpression of SIRT-1 significantly improved cardiac function by decreasing ferroptosis and reducing ROS levels through upregulation of SLC7A11 protein expression and downregulation of p53 protein expression [50]. These findings indicate that the crosstalk between ROS and the SIRT-1/p53/SLC7A11 pathway is critical for the inhibition of MI/RI by ferroptosis.

In a rat model of ischemia/reperfusion, GPX4 levels were lower, but the levels of iron, ACSL4, and malondialdehyde were higher upon prolonged reperfusion [51]. Overexpression of mitochondrial GPX4 can preserve cardiac function in ischemia/reperfusion injury by reducing lipid peroxidation [52]. Furthermore, the ferroptosis inhibitor Lip-1 is a cardioprotective factor for ischemia/reperfusion by increasing GPX4 level to inhibit ferroptosis and lipid production [49]. These findings suggest that targeting GPX4 expression in the heart can significantly reduce ferroptosis damage in mouse hearts with MI/RI. A recent study reported that mitochondrial dysfunction and damage caused by ischemia/reperfusion were markedly alleviated after dexmedetomidine administration prior to occlusion of the left anterior descending artery occlusion and reperfusion. The alleviation involved reversal of the inhibited activity of SLC7A11/GPX4 [17]. These findings demonstrate that DEX inhibits ferroptosis by enhancing the expressions of SLC7A11 and GPX4, which are key factors in attenuating ischemia/reperfusion injury. Hence, GPX4 may be a potential target for the treatment of MI/RI. A growing number of studies have shown that ferroptosis in cardiomyocytes can be regulated by autophagy in animal models of MI/RI. The molecular mechanisms of ferroptosis mainly involve mammalian target of rapamycin (mTOR) and Beclin1. Moreover, mTOR exerts its functions in myocardial ischemia both by the AMP-activated protein kinase/mTOR and phosphatidylinositol-3-kinase/Akt/mTOR signalling pathways [ 53, 54] . Strikingly, ELAV-like RNA binding protein 1 (ELAVL1) is required to execute cardiomyocyte autophagy and ferroptosis by specifically binding to the autophagy-associated protein Beclin1, which facilitates a decrease in p62 protein expression and an increase in LC3 level in an animal model of MI/RI. However, inhibition of ELAVL1-mediated autophagic cell death (also known as ferritinophagy) leads to resistance of cardiac myocytes to ferroptosis-induced injury [55], implying that inhibiting the autophagic regulation of ferroptosis is a promising target for the treatment of MI/RI.

### Ferroptosis and heart failure

HF is increasingly being recognised as a common, complex, and heterogeneous clinical syndrome. Clinical studies have shown that iron homeostasis is important for the maintenance of cardiomyocyte function. However, disruption of iron homeostasis due to iron deficiency or iron overload contributes to heart failure (HF).

Mice lacking ferritin H display decreased Slc7a11 expression and increased ROS production, eventually leading to the occurrence of HF [56]. Furthermore, overexpression of cardiomyocyte-specific SLC7A11 leads to an increase in GSH level, resulting in reduced cardiac ferroptosis and partial HF. This highlights the importance of ferritin-mediated ferroptosis in HF [56].

Recently, *in vitro* experiments have shown that diabetes-induced HF in mice overexpressing Nr2f2 (AAV9-cTNT-Nr2f2) resulted in severe HF, ferroptosis, mitochondrial dysfunction and a strong oxidative stress response compared with control mice [57]. Meanwhile, *in vivo* studies have shown that Nr2f2 promotes HF and ferroptosis in diabetes-induced HF by regulating PGC-1α signalling [57]. These results indicate that Nr2f2 plays a key regulatory role in the pathological mechanism of diabetes-induced HF, thus highlighting a new potential target for the treatment of diabetes-induced HF.

In addition, the circular RNA circSnx12, which directly targets miR-224-5p, has been implicated in the pathogenesis of ferroptosis during HF [58], indicating that circular RNA may be a potential biomarker or therapeutic target for the treatment of HF. In addition, puerarin, acting as an antioxidant regulator, can protect against myocyte loss and partially alleviate HF by significantly blocking lipid peroxidation and ferroptosis [59]. Furthermore, knockdown of Toll-like receptor 4 ( *TLR4*) and NADPH oxidase 4 ( *NOX4*) by transfection with lentiviral small interfering RNAs in cardiac muscle has been reported to exert an inhibitory effect on ferroptosis and reduce myocyte loss. Negative regulation of the TLR4-NOX4 axis may alleviate HF by abrogating ferroptosis. Hence, the TLR4-NOX4 axis may be a potential therapeutic target for HF [60]. Collectively, these findings indicate that ferroptosis plays a key role in HF and that targeted inhibition of ferroptosis may be a new approach for the treatment of HF.

### Ferroptosis and atherosclerosis

Atherosclerosis (AS) is the underlying cause of most CVDs. AS itself is a pathological disease characterised by dysregulation of lipid metabolism [61]. Lipid peroxidation is a critical driver of ferroptosis, and overexpression of GPX4 prevents progression of AS by blocking lipid peroxidation. Ferroptosis may therefore have a significant impact on AS [62]. Fer-1 suppresses high-fat diet-induced AS in ApoE mice by upregulating SLC7A11 and GPX4 levels, thereby reducing iron levels and lipid peroxidation [63]. In addition, the expressions of PTGS2, ACSL4, GPX4, and NLR family pyrin domain containing 3 (NLRP3) are upregulated, but the expression of GPX4 is downregulated, in the coronary arteries of patients with advanced AS [ 3, 64] . These findings suggest that ferroptosis modulates the onset and progression of AS and that SLC7A11 and GPX4 may be potential targets for the prevention and treatment of coronary AS.

## Ferroptosis Intervention in CVDs

Ferroptosis is a promising target for the prevention and treatment of CVDs. This section details ferroptosis-associated targets and drugs.

### Iron metabolism-associated targets and drugs

#### Inhibition of iron overload: dexrazoxane, deferoxamine, deferiprone

Dexrazoxane, a cyclic derivative of ethylenediaminetetraacetic acid, is currently used as an antitumor adjunct to reduce myocardial toxicity induced by chemotherapy with anthracyclines, such as doxorubicin [65]. Interestingly, dexrazoxane protects against doxorubicin-induced cardiac injury by inhibiting ferroptosis, but other iron chelators do not appear to be effective against doxorubicin-induced cardiac injury [66]. The reason for this is that dexrazoxane readily enters the mitochondria of cardiomyocytes by crossing the cell membrane, and it reduces the accumulation of iron by chelating the free iron in the cell, while other iron complexing agents cannot enter the mitochondria [67]. In addition, deferoxamine and deferiprone alleviate septic cardiomyopathy and MI/RI by blocking ferritin [ 45, 51] .

#### Inhibition of mitochondrial iron overload: Mito-FerroGreen, Ru360, cyclosporine A

Mito-FerroGreen is a novel mitochondria-specific iron chelator that can prevent and alleviate doxorubicin-induced cardiomyopathy by inhibiting mitochondrial iron overload [68]. Ru360 is a highly specific inhibitor of the mitochondrial calcium uniporter (MCU) [69]. It has been shown that Ru360 prevents iron-overload cardiomyopathy by inhibiting iron uptake into cardiac mitochondria [70]. Cyclosporine A is an inhibitor of the mitochondrial permeability transition pore that can alleviate the proarrhythmic effects of iron overload [71].

#### Inhibition of ferritinophagy without damaging general autophagy pathways

Previous studies have shown that ferritinophagy plays an indispensable role in CVDs caused by ferroptosis. Therefore, it is particularly important to study the targets and drugs related to ferritinophagy [72]. NCOA4 has been shown to selectively transport ferritin to lysosomes, eventually causing ferritinophagy [73]. Moreover, some inhibitors of NCOA4 can inhibit ferritinophagy without hindering general autophagy pathways. For example, cyanidin-3-glucoside (C3G) can alleviate ferroptosis and MI/RI damage by inhibiting ferritinophagy [74]. Hence, C3G appears to be a promising therapeutic option for MI/RI. Furthermore, inhibition of DNMT-1 could provide protection against diabetic MI/RI by lowering the levels of DNMT-1, NCOA4, and ferroptosis [75]. The inhibition of DNMT-1 is mainly through regulation of NCOA4-mediated ferritinophagy. The first NCOA4-FTH1 interaction inhibitor, designated compound 9a, has recently been reported [76]. Compound 9a reduces ferroptosis by disrupting the NCOA4-FTH1 interaction to reduce cellular ferrous iron level [76]. Further research showed that compound 9a can significantly ameliorate MI/RI in rats.

Polymer nanoparticles have been reported to effectively enhance the brain delivery of resveratrol, thus allowing intracerebral haemorrhage to be treated by inhibition of erastin-induced ferroptosis in mouse hippocampal cells [77]. Other types of nanoparticles could enhance ferroptosis in cancer cells by promoting GSH depletion and lipid peroxidation [78]. Nanoparticles may, therefore, serve as potential treatments for cardiovascular disease caused by ferroptosis and ferritinophagy, although nanoparticles can also have effects on the gastrointestinal microbiota and exhibit potential toxicity in different organs and body systems [79].

### Lipid peroxidation-associated targets and drugs

#### Inhibition of lipid peroxidation: Ferrostatin 1 and liproxstatin 1

Ferrostatin 1 is the first specific and potent inhibitor of ferroptosis [1]. It has been shown that ferrostatin 1 protects against doxorubicin-induced cardiomyopathy and prevents doxorubicin-induced cardiomyocyte death [ 38, 68] . In addition, ferrostatin 1 has been shown to improve cardiac function in animal models with MI/RI [ 42, 80] . In addition, it has been recently reported that ferrostatin 1 attenuates sepsis-induced cardiomyopathy and atherosclerosis in mice [ 45, 63] .

Liproxstatin 1, a spiroquinoxalinamine derivative, is also a strong inhibitor of ferroptosis [81]. Previous studies have shown that Lip-1 induces cardioprotective effects against I/R injury by reducing mitochondrial ROS production and maintaining GPX4 activity [82].

#### Inhibition of glutaminolysis: Compound 968, Xanthohumol, Qing-Xin-Jie-Yu Granule, britanin

Compound 968 is a cell-permeable inhibitor of glutaminolysis. Compound 968 alleviates MI/RI by targeting glutaminolysis to inhibit ferroptosis [8]. Xanthohumol (XN), a principal prenylflavonoid, has anti-inflammation and anti-oxidant activities [83]. Previous studies have shown that XN alleviates the ischemia-reperfusion injury by regulating the protein level of GPX4 [83]. Qing-Xin-Jie-Yu Granule is a traditional Chinese medicine formula used in clinical treatment of atherosclerosis [84]. Qing-Xin-Jie-Yu Granule was recently shown to result in inhibition of ferroptosis and stabilization of atherosclerotic plaques partially by adjusting the GPX4/xCT signaling pathway [85]. Britanin has a variety of pharmacological effects, such as antitumor and anti-inflammatory activities [86]. What is more, it can alleviate ferroptosis-mediated the myocardial ischaemia/reperfusion injury via regulating the level of GPX4 [86].

#### Glutathione precursor: N-Acetyl cysteine

*N*-Acetyl cysteine, an antioxidant and glutathione precursor, is responsible for enhancing the endogenous synthesis of glutathione [87]. A growing number of studies have shown that *N*-acetyl cysteine exerts a protective effect on CVD and substantially reduces MI/RI caused by ferroptosis in diabetic rats [88].

### FUNDC1 and CD74 ablation

FUN14 domain-containing protein 1 (FUNDC1), a novel mitophagy receptor, has been implicated in the pathogenesis of several diseases, including cardiovascular disease [89]. FUNDC1 ablation was recently shown to result in inhibition of paraquat-induced mitochondrial damage, ferroptosis, and cardiomyocyte contractile dysfunction, and the effects were abrogated by inhibition of JNK [90]. Interestingly, FUNDC1 deficiency has been shown to sensitise cardiac remodelling and dysfunction via ACSL4-mediated ferroptosis [91].

CD74, a protein trafficking regulator, is the receptor of macrophage migration inhibitory factor [92]. Its ablation has been shown to attenuate type 2 diabetes mellitus-induced cardiac remodelling and systolic dysfunction by inhibiting NLRP3-caspase-1 activation, oxidative stress, lipid peroxidation and ferroptosis [93].

### Other targets and drugs

In addition to the above-mentioned targets and drugs, other drugs and targets can also lead to inhibition of cardiac disease by ferroptosis. For example, dexmedetomidine, an α2-adrenergic receptor agonist, can activate GPX4. Dexmedetomidine alleviates sepsis-induced cardiomyocyte injury in mice by activating the ferroptosis inhibitor GPX4 [94]. Vas2870, a novel Nox2 inhibitor, inhibits Nox2 expression *in vivo* and *in vitro* and protects diabetic rats from myocardial MI/RI [88]. Moreover, P22077, an inhibitor of ubiquitin-specific protease 7, can inhibit the p53–TFR1 pathway. P22077 can inhibit ferroptosis in rats by reducing p53 and TFR1 levels, thus providing protection against MI/RI [95]. Sulforaphane, an adenosine modulator, can activate NRF2 [96]. Previous studies have shown that sulforaphane prevents ferroptosis and MI/RI from occurring in diabetes through AMPK-mediated NRF2 activation [5]. Acadesine, also known as 5-aminoimidazole-4-carboxamide (AICA) riboside, can protect the myocardium by activating adenosine monophosphate-activated protein kinase (AMPK). A growing number of studies have shown that acadesine increases the heart’s ability to recover from ischemia and reperfusion by inhibiting ferroptosis [97]. Interestingly, zinc protoporphyrin IX, a metalloporphyrin, is a competitive inhibitor of HO-1 [98]. It has been shown that zinc protoporphyrin IX reduces ferroptosis induced by doxorubicin in mice by blocking heme degradation and free iron release [38]. In addition, salubrinal, an ER stress inhibitor, alleviates MI/RI by selectively inhibiting eIF2-alpha dephosphorylation, which results in inhibition of ER stress [ 99, 100] , thereby providing another potential therapeutic strategy for the prevention of MI/RI. Furthermore, puerarin, an isoflavone extracted from the kudzu root, is used as a conventional cardiovascular drug in countries in eastern Asia [101]. Puerarin has been shown to improve end-stage CVD (HF) by inhibiting myocyte loss and ferroptosis [59].

### Risk of using medications

Although a variety of targets and drugs that inhibit ferroptosis have been confirmed to alleviate CVD, the drugs mentioned above may also be associated with a degree of potential risk. For example, early studies have reported that deferoxamine has toxic effects on the retina when used at levels above the therapeutic index [102]. Arthralgia is more frequent in patients taking deferiprone [103]. Moreover, the risk of adverse events associated with the combination of deferiprone and deferoxamine was significantly increased [103]. A number of studies have shown that the incidences of various side effects increase as the dose of *N*-acetyl cysteine increases [104]. Furthermore, patients undergoing cardiac surgery treated with dexmedetomidine are prone to overall hypotension or bradycardia [105]. High doses of sulforaphane produce hypothermia, impaired motor coordination, decreased skeletal muscle strength and even death [106]. Moreover, it has been shown that high doses of puerarin increase the risk of endometrial hyperplasia in healthy women [107]. Therefore, there are significant challenges and risks associated with the application of puerarin.

## Outlook and Future Directions

This article provides a review of the genes and pathways involved in ferroptosis, including iron metabolism, lipid peroxidation, the GSH system, and the BH4/CoQ10 system. Ferroptosis is a recently recognised form of cell death that is widely involved in the pathogenesis of various diseases. It plays a dual role in disease pathogenesis. However, there is a lack of reliable and sensitive biomarkers and probes for ferroptosis. Thus, additional biomarkers and probes need to be explored for better assessment of ferroptosis. Finally, the clinical relevance of ferroptosis in CVD has not been clearly established, thus warranting further assessment via clinical trials. The scientific details and issues discussed in this review will provide a better understanding of the exact role of ferroptosis in various types of CVD.
